# Colistin Resistance Mediated by *mcr-1* in ESBL-Producing, Multidrug Resistant *Salmonella* Infantis in Broiler Chicken Industry, Italy (2016–2017)

**DOI:** 10.3389/fmicb.2018.01880

**Published:** 2018-08-17

**Authors:** Virginia Carfora, Patricia Alba, Pimlapas Leekitcharoenphon, Daniele Ballarò, Gessica Cordaro, Paola Di Matteo, Valentina Donati, Angela Ianzano, Manuela Iurescia, Fiorentino Stravino, Tania Tagliaferri, Antonio Battisti, Alessia Franco

**Affiliations:** ^1^National Reference Laboratory for Antimicrobial Resistance, Istituto Zooprofilattico Sperimentale del Lazio e della Toscana “M. Aleandri,” General Diagnostics Department, Rome, Italy; ^2^European Union Reference Laboratory for Antimicrobial Resistance, WHO Collaborating Centre for Antimicrobial Resistance in Foodborne Pathogens and Genomics, National Food Institute, Technical University of Denmark, Kongens Lyngby, Denmark

**Keywords:** colistin resistance, *mcr* genes, ESBL (Extended Spectrum Beta-Lactamases), plasmids, whole genome sequencing, *Salmonella* Infantis, broilers, broiler meat

## Abstract

Colistin-resistance mediated by mobilisable and plasmid-borne *mcr* genes has emerged worldwide, threatening the efficacy of colistin, a last resort antibiotic increasingly used for treating human invasive infections by multidrug-resistant or extensively drug-resistant *Enterobacteriaceae*. In this study, we report the first evidence of *mcr*-1-mediated colistin resistance in four multidrug resistant (MDR) out of 324 *Salmonella* infantis from the Italian antimicrobial resistance (AMR) monitoring (2001–2017) in broilers and broiler meat. Two were also Extended Spectrum Beta-Lactamases (ESBL)-producing isolates. Characterization by whole genome sequencing (WGS), located *mcr*-1.1 on an incX4 plasmid. Phylogenetic analysis of these isolates with selected Italian *S*. Infantis previously isolated from animals, meat and human clinical cases with unknown epidemiological relationship, demonstrated that ESBL-producing, *mcr*-1-positive isolates belonged to the emerging pESI-like-positive-ESBL-producing clone described in Italy in 2015.

## Introduction

Colistin-resistance mediated by mobilisable and plasmid-borne *mcr* genes, has emerged worldwide in humans and food-producing animals, threatening the efficacy of colistin, a last resort antibiotic of the polymyxin family, increasingly used for treating human invasive infections by multidrug-resistant or extensively drug-resistant *Enterobacteriaceae* (Poirel et al., 2017). *Salmonella enterica* serovar Infantis represent one of the top five *Salmonella* serovars involved in human infections in Europe and the most frequent serovar detected in broilers (45.6%) and broiler meat (47.4%) (EFSA, 2017). The increasing incidence of *S*. Infantis infections may be complicated by the spread of MDR strains, such as the recent spread of MDR, ESBL-producing *S*. Infantis in broiler chickens, broiler meat and humans. It is characterized by the presence of a conjugative pESI-like megaplasmid, firstly described in Israel in 2014 (Aviv et al., 2014), and then in Italy in 2015 (Franco et al., 2015) and more recently reported in Switzerland (Hindermann et al., 2017) and United States (Tate et al., 2017).

In this study we report the first evidence of *mcr*-1-mediated colistin resistance in four multidrug resistant (MDR) *S*. Infantis, with two of them being also extended-spectrum cephalosporin-resistant (ESC-R) and ESBL-producing, isolated from broilers and broiler meat samples in the frame of the Italian antimicrobial resistance (AMR) monitoring. The four isolates were in-depth characterized by whole genome sequencing (WGS) and bioinformatics analysis, including phylogenetic relationships with previously characterized Italian *S*. Infantis belonging to the pESI-like positive, Extended Spectrum Beta-Lactamases (ESBL)-producing clone emerged in Italy.

## Materials and methods

### Isolates

Four multidrug resistant (MDR) *S*. Infantis, displaying a colistin MIC value ≥ 4 mg/L, were detected among 324 *S*. Infantis isolates collected in the frame of antimicrobial resistance (AMR) monitoring activities conducted from 2001 to 2017 by the National Reference Laboratory for Antimicrobial Resistance (NRL-AR) and screened for antimicrobial susceptibility. The four *S*. Infantis isolates originated from broilers (*n* = 2) and broiler meat samples (*n* = 2) (Supplementary Table 1).

### Antimicrobial susceptibility testing of *Salmonella* isolates

Antimicrobial Susceptibility testing was performed as minimum inhibitory concentration (MIC) determination by broth microdilution, using the EU consensus 96-well microtitre plates (Trek Diagnostic Systems, Westlake, OH, USA). The results were interpreted according to epidemiological cut-offs included in the Annex A of the EU Decision 2013/652/EU^1^. *Escherichia coli* ATCC 25922 was used as Quality Control strain.

### Molecular characterization

PCR-screening of *mcr* genes groups (*mcr*-1, *mcr*-2, *mcr*-3, *mcr*-4, and *mcr*-5) in all the four *S*. Infantis and ESBL/AmpC genes in the two ESC-R isolates (16092401-41 pESI IncX4 and 16092401-42 pESI IncX4, Table 1), was performed as previously reported (Franco et al., 2015; Rebelo et al., 2018).

**Table 1:** Genomic and phenotypic characteristics of the four colistin-resistant *S*. Infantis isolates analyzed by WGS.

**Isolate ID**	**Origin**	**Year of isolation**	**ENA accession number**	**ST**	**Antimicrobial resistance profile**			**Virulence, colonization, and enhanced fitness genes**	**Plasmid content**	
					**Chromosomal point mutations**	**Horizontally acquired genes**	**Phenotypic AMR profile**		**Plasmid replicons**	**IncI1 Plasmid MLST (pMLST)**
16092401-41 pESI IncX4	Broiler chicken	2016	ERS2521096	32	*gyr*A p.D87G	*aph(3′), bla*_CTX−M−1_*, mcr-1.1, sul1, tet*(A), *dfrA1, dfrA14*	AMP- CAZ CTX - CIP- COL- NAL- SMX- TET- TMP	*K88, fim, faeD, ipf, irp2, qacEΔ, mer* operon, *ccdB/ccdA* _toxin/antitoxin, *pemK/pemI* toxin/antitoxin, *hicB/hicA* toxin/antitoxin	IncP^*a*^ [pESI backbone (*bla*_CTX−M−1_)], IncX4 (*mcr*-1.1)	*ardA_*2*, pilL_*3*, sogS_*9*, trbA_*21
16092401-42 pESI IncX4	Broiler chicken	2016	ERS2521097	32	*gyr*A p.D87G	*aph(3′), bla*_CTX−M−1_*, mcr-1.1, sul1, tet*(A), *dfrA1, dfrA14*	AMP- CAZ- CTX- CIP- COL-NAL-SMX- TET- TMP	*K88, fim, faeD, ipf, irp2, qacEΔ, mer* operon, *ccdB/ccdA* toxin/antitoxin, *pemK/pemI* toxin/antitoxin, *hicB/ hicA*_toxin/antitoxin	IncP^*a*^ [pESI backbone (*bla*_CTX−M−1_)], IncX4 (*mcr*-1.1)	*ardA_*2*, pilL_*3*, sogS_*9*, trbA_*21
16072017 pESI IncX4	Broiler chicken	2016	ERS2030111	32	*gyr*A p.D87G	*aadA1, aadA2, bla_*TEM*−1*B*_, mcr-1.1, cmlA1, sul1, sul3, tet*(A), *dfrA14*	AMP- CIP- CHL- COL- NAL- SMX- TET- TMP	*K88, fim, faeD, ipf, irp2, qacEΔ, mer* operon, *ccdB/ccdA* toxin/antitoxin, *pemK/pemI* toxin/antitoxin, *hicB/ hicA* toxin/antitoxin	IncP^*b*^ (pESI backbone), IncX4 (*mcr*-1.1)	*ardA_*2*, pilL_*3*, sogS_*9*, trbA_*21
17095712-68 pESI IncX4	Broiler meat	2017	ERS2521098	32	*gyr*A p.D87G	*aadA1, aadA2, bla_*TEM*−1*B*_, mcr-1.1, cmlA1, sul1, sul3, tet*(A), *dfrA14*	AMP- CIP- CHL- COL- NAL- SMX- TET- TMP	*K88, fim, faeD, ipf, irp2, qacEΔ, mer* operon, *ccdB/ccdA* toxin/antitoxin, *pemK/pemI* toxin/antitoxin, *hicB/ hicA* toxin/antitoxin	IncP^*b*^(pESI backbone), IncX4 (*mcr*-1.1), IncX1ColRNAI	*ardA_*2, *pilL_*3, *sogS_*9*, trbA_*21

*ENA, European Nucleotide Archive; ST, Sequence Type following the scheme of Enterobase^8^; AMR, Antimicrobial Resistance; AMP, ampicillin; CAZ, ceftazidime; CTX, cefotaxime; CIP, ciprofloxacin; CHL, chloramphenicol; COL, colistin; NAL, nalidixic acid; SMX, sulfamethoxazole; TET, tetracycline and TMP, trimethoprim*.

a *tested positive for oriV, Plasmid RK2 (from E.coli) DNA with transposon (Tn1723) insertion sites, DNA_rep*.

b *tested positive for oriV, Plasmid RK2 (from E.coli) DNA with transposon (Tn1723) insertion sites, DNA_rep, trfA2*.

For WGS, the genomic DNA was extracted using the QIAamp DNA Mini Kit (Qiagen, Hilden, Germany) following the manufacturer's protocol. Libraries were prepared for Illumina pair-end sequencing using the Illumina (Illumina, Inc., San Diego, CA) NexteraXT® Guide 150319425031942 and sequenced in a MiSeq sequencer (Illumina platform). Raw sequence data of the four isolates were submitted to the European Nucleotide Archive^2^ under the accession numbers reported in Table 1.

Raw reads quality was improved by trimming with TrimmomaticPE v0.22 (Bolger et al., 2014) with the following parameters: Q30 as minimum quality required for maintaining a base from the beginning and from the end of the read and windows size of 10 with Q20 as average quality. Processed reads were “*de novo*” assembled using SPAdes v3.11.0 (Nurk et al., 2013) with the default parameters and in parallel with the plasmid-option in order to obtain only contigs from the present plasmids. Molecular characterization was performed by analyzing the assemblies with different bioinformatics tools: CGE tools for the seven genes Multilocus Sequence Typing (MLST 1.8^3^) to assign Sequence Types (STs), ResFinder 3.0^4^ (Zankari et al., 2012) for the genetic basis of AMR; BLAST v2.2.31 (Zhang et al., 2000) for the identification of plasmid incompatibility groups, plasmidMLST, pESI-like markers, fitness and virulence genes, using CGE^5^ and Genbank (Supplementary Table 2) databases as references.

Identification of important mutations in CcdB (toxin-antitoxin system) was carried out by comparing the *ccdB* gene of each isolate against the Genbank database using BLAST on-line tool and compared with the aminoacid sequence of the CcdB reference protein from *E. coli* and *Salmonella spp*. (Di Cesare et al., 2016).

**Figure 1:**
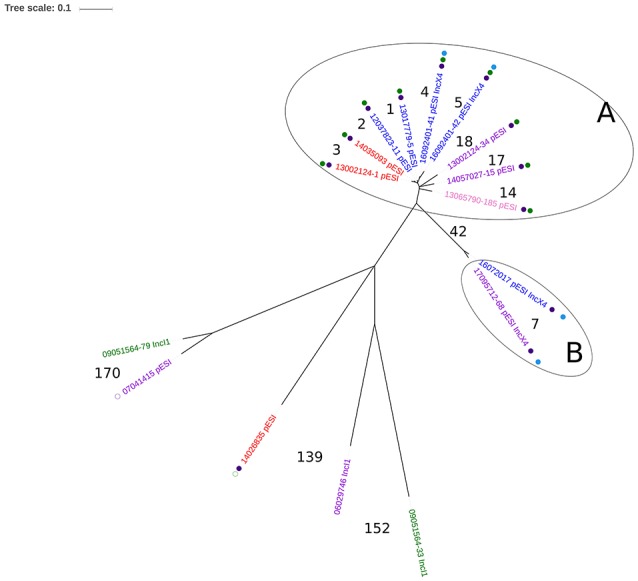
Single-nucleotide polymorphism (SNP)-based phylogeny of 16 selected ESC-resistant, ESBL-producing, and ESC-susceptible *Salmonella* Infantis mainly from broiler chicken, broiler meat, and humans in Italy (2006–2017). Colors of the isolate ID indicate the sample host: red: human; purple: broiler meat; blue: broiler chicken; pink: pig; green: guinea fowl. Full purple dot: presence of the mutation D87G in the *gyr*A gene; empty purple dot: presence of the mutation S83Y in the *gyr*A gene. Full green dot: presence of *bla*_CTX−M−1_ ESBL gene; Empty green dot: presence of *bla*_CTX−M−65_ ESBL gene. Full light blue dot: presence of *mcr*-1.1. Numbers in the figure indicate the number of SNPs difference between two isolates or between clusters. Two clusters were identified based on the number of SNPs: Cluster A, with a difference of 1-18 SNPs between the isolates, and Cluster B with a difference of 7 SNPs between the isolates. Isolates of Cluster A and Cluster B differed at least by 42 SNPs. Details of the 16 isolates are indicated in the Supplementary Table 1.

A Single-Nucleotide Polymorphisms (SNPs) tree was built using CSI Phylogeny 1.4^6^ (Kaas et al., 2014). Basically, raw reads from the four isolates and 12 *S*. Infantis previously studied (Franco et al., 2015) were aligned against the reference genome *S*. Infantis SINFA (LN649235), using BWA v. 0.7.2 (Li and Durbin, 2009). The depth at each mapped position was calculated using genomeCoverageBed (BEDTools v. 2.16.2), (Quinlan and Hall, 2010). SNPs were called using “mpileup” (SAMTools v. 0.1.18) (Li et al., 2009). SNPs were filtered out if the depth at the SNP position was not at least 10x or at least 10% of the average depth for the particular genome mapping and if the mapping quality was below 25 or the SNP quality was below 30, calculated by BWA and SAMTools, respectively. The pruning distance was set at 10 bp. Then, all genome mappings were compared and all positions where SNPs was called in at least one mapping were validated in all mappings and ignored if fails validation. The validation includes both depth and Z-score for the SNP filtering. Maximum Likelihood tree was created using FastTree (Price et al., 2010), based on a total of 412 informative SNPs (Figure 1) and edited with iTOL^7^ (Letunic and Bork, 2016). Clusters separation was performed as previously described (Kaas et al., 2014; Franco et al., 2015).

## Results

### Antimicrobial susceptibility testing of salmonella isolates

All the four isolates were colistin-resistant (MIC value ≥ 4 mg/L), MDR and two of them also ESC-R (both isolates displayed cefotaxime and ceftazidime MICs values = 32 mg/L and 4 mg/L, respectively) (Table 1). All displayed fluoroquinolone microbiological resistance (MIC 0.25 mg/L).

### Molecular characterization

PCR-screening of *mcr* genes groups revealed that all isolates were *mcr*-1 positive, while the two ESC-R isolates tested positive also for *bla*_CTX−M−1_ gene.

Details of genomic characteristics of the four isolates analyzed by WGS are reported in Table 1. All the four *S*. Infantis, belonged to the Sequence Type (ST) ST32 and presented the same point mutation (D87G) in *gyr*A (Table 1) associated with fluoroquinolones resistance, similarly to the previously characterized Italian pESI-like-positive ESC-R isolates (Figure 1) (Franco et al., 2015). All isolates harbored: a pESI-like plasmid, characterized by the presence of *ori*V from IncP, the ardA 2, pilL 3, sogS 9, and trbA 21 pMLST alleles of the IncI1 plasmid, genes coding for different toxin-antitoxin systems (CcdB/CcdA and PemK/PemI) and the specific markers associated with virulence, enhanced colonization capability and enhanced fitness previously described in pESI-like-positive S. Infantis in Italy (Franco et al., 2015) (Table 1); an IncX4 plasmid harboring the *mcr*-1.1 variant and the genes coding for the HicAB toxin-antitoxin complex, located in the same contig.

The study of the protein CcdB, from the above described toxin-antitoxin system, revealed that all four isolates had the same aminoacid sequence and presented a tryptophan aminoacid in position 99, as the *E. coli* CcdB reference.

Regarding the acquired AMR profiles, all presented a very similar AMR accessory gene content (Table 1). All displayed resistance to colistin mediated by the *mcr*-1.1 variant and resistance to tetracycline, sulfamethoxazole and trimethoprim mediated by the pESI-like borne *tet*(A), *sul*1 and *dfr*A14 genes. The two ESC-R isolates presented the same phenotype and harbored *bla*CTX-M-1 in pESI-like plasmid-derived sequences according to the plasmid-SPAdes output. Similarly, the two ESC-S isolates presented the same gene content including the *cmlA1* gene, mediating chloramphenicol resistance (Table 1).

Regarding the phylogenetic analysis, the two ESC-R, ESBL-producing (blaCTX-M-1), *mcr*-1-positive, *S*. Infantis (isolates 1 and 2) were grouped in the same cluster (Figure 1, Cluster A), differing only by 4 to 18 SNPs from seven pESI-like-positive, *bla*_CTX−M−1_ *S*. Infantis belonging to the emerging, ESBL-producing clone mainly detected in the Italian broiler chicken industry and infecting humans (Franco et al., 2015). The two ESC-S, *mcr*-1-positive, *S*. Infantis were part of a different cluster (Figure 1, Cluster B), being 42 SNPs the minimum difference with isolates of Cluster A. The isolates of Cluster A were separated at least by 109 SNPs from the remaining five isolates, four representing earlier animal strains circulating in Italy, and one single human *bla*_*CTX*__−M−65_-positive clinical isolate detected in a patient hospitalized in Italy in 2014 (Franco et al., 2015).

## Discussion

In the present study, we report for the first time the isolation and characterization of four MDR *S*. Infantis containing both pESI-like megaplasmid and IncX4 plasmid harboring *mcr*-1.1. Additionally, two of them were ESBL producers and, as already previously demonstrated (Franco et al., 2015), the *bla*_CTX−M−1_ gene was located in pESI-like plasmids, as confirmed by comparing their contigs containing *bla*_CTX−M−1_ with the same plasmid region of the eight ESBL-producing (CTX-M-1 type), pESI-like-positive *S*. Infantis previously investigated and reported in Italy (Franco et al., 2015). The two isolates were also within the same cluster (4–18 SNPs difference) that included isolates from broiler chicken, broiler meat and human clinical cases with unknown epidemiological relationship, all belonging to the ESBL-pESI-like-positive *S*. Infantis clone previously described in Italy (Franco et al., 2015). The four isolates also presented plasmidic genes coding for multiple Type II toxin/antitoxin modules, as already reported for clones of other virulent serovars of *S. enterica*, differently from less-pathogenic ones which harbor none or low numbers of these genetic elements (De la Cruz et al., 2013; Lobato-Márquez et al., 2015). Previous studies have demonstrated the central role of these gene loci in bacterial adaptability in response to stress conditions and in the maintenance of plasmids or genomic islands (Goeders and Van Melderen, 2014), therefore, in pathogenic Salmonellas they could contribute to enhancing their fitness inside eukaryotic cells, also supporting the ecological success of certain clones, such as the pESI-like-positive-ESBL-producing *S*. Infantis clone, as described by Aviv et al. (2014). In particular, the CcdA-CcdB complex has been reported to contribute to the maintenance of plasmids or genomic islands by activation of post-segregational killing mechanisms of the cell (Goeders and Van Melderen, 2014), unless specific amino acid substitutions in the *ccdB* sequence known to compromise *in vitro* the lethal effect of CcdB in the absence of antitoxin CcdA, are present (Lobato-Márquez et al., 2015). Differently from other *Salmonella* serovars (Di Cesare et al., 2016), in this study, the sequence analysis of the *ccdB* gene in all the four isolates revealed the presence of the tryptophan residue in position 99, described as essential for the toxicity of CcdB in *E. coli* (Loris et al., 1999).

Overall, these findings are of great concern, since this clone, has genetic traits of enhanced virulence, MDR and fitness in the intensive farming system, and it is often ESBL-producing (all traits mediated by the conjugative pESI-like plasmid). Additionally, we have demonstrated in this study that it has the attitude to acquire additional extra-chromosomal, transferable resistance to last-resort drugs like colistin (Poirel et al., 2017). As resulted from the WGS analysis, the characterized *mcr*-1.1 gene was harbored by another conjugative plasmid (IncX4), already involved in transferable *mcr*-mediated colistin resistance in *Enterobacteriaceae* and other *Salmonella* serovars in Italy (Carattoli et al., 2017; Alba et al., 2018) and other EU countries (Borowiak et al., 2017; Garcia-Graells et al., 2018). These characteristics inevitably lead to a further reduction of therapeutic options for invasive infections in humans, including in case of ESBL-producing, MDR *Salmonella* strains transmitted through the food chain. Our results highlight the need of implementation of risk-management strategies and actions to be taken in order to: a. drastically reduce the amount of colistin used in broilers in Italy as recommended in food-producing animals by the European Medicines Agency (EMA, 2016: target: below 5 mg/PCU, and ideally at 1 mg/PCU); b. reduce the overall prevalence of *S*. Infantis, and especially of the new emerging ESBL-producing, MDR clone, within the Italian broiler chicken industry.

## Author contributions

AB, AF, PA, VC, PL conceived and designed the experiments. AI, MI, FS, GC, PD, DB, TT, PA, PL, VD performed the experiments. PA, VC, PL, VD, AI, MI, FS, GC, PD, DB, TT analyzed the data. AB, AF, PL contributed reagents, materials, analysis tools. VC, PA, AB, AF wrote the paper. All authors contributed to manuscript revision, read and approved the submitted version.

### Conflict of interest statement

The authors declare that the research was conducted in the absence of any commercial or financial relationships that could be construed as a potential conflict of interest.

## Footnotes

^1^http://eur-lex.europa.eu/legal-content/EN/TXT/HTML/?uri=CELEX:32013D0652&from=IT.

^2^http://www.ebi.ac.uk/ena.

^3^https://cge.cbs.dtu.dk/services/MLST/.

^4^https://cge.cbs.dtu.dk/services/ResFinder/.

^5^https://cge.cbs.dtu.dk/services/PlasmidFinder/.

^6^https://cge.cbs.dtu.dk/services/CSIPhylogeny/.

^7^http://itol.embl.de/.

^8^https://enterobase.warwick.ac.uk/.
