# Corrigendum: Colistin Resistance Mediated by *mcr-1* in ESBL-Producing, Multidrug Resistant *Salmonella* Infantis in Broiler Chicken Industry, Italy (2016–2017)

**DOI:** 10.3389/fmicb.2018.02395

**Published:** 2018-10-08

**Authors:** Virginia Carfora, Patricia Alba, Pimlapas Leekitcharoenphon, Daniele Ballarò, Gessica Cordaro, Paola Di Matteo, Valentina Donati, Angela Ianzano, Manuela Iurescia, Fiorentino Stravino, Tania Tagliaferri, Antonio Battisti, Alessia Franco

**Affiliations:** ^1^National Reference Laboratory for Antimicrobial Resistance, Istituto Zooprofilattico Sperimentale del Lazio e della Toscana “M. Aleandri, ” General Diagnostics Department, Rome, Italy; ^2^European Union Reference Laboratory for Antimicrobial Resistance, WHO Collaborating Centre for Antimicrobial Resistance in Foodborne Pathogens and Genomics, National Food Institute, Technical University of Denmark, Kongens Lyngby, Denmark

**Keywords:** colistin resistance, *mcr* genes, ESBL (Extended Spectrum Beta-Lactamases), plasmids, whole genome sequencing, *Salmonella* Infantis, broilers, broiler meat

In the original article, there was an error in the Materials and Methods, subsection Isolates. The four *S*. Infantis isolates originated from broilers (*n* = 2) and broiler meat samples (*n* = 2).

A correction has been made to Materials and Methods, subsection Isolates: Four multidrug resistant (MDR) *S*. Infantis, displaying a colistin MIC value ≥ 4 mg/L, were detected among 324 *S*. Infantis isolates collected in the frame of antimicrobial resistance (AMR) monitoring activities conducted from 2001 to 2017 by the National Reference Laboratory for Antimicrobial Resistance (NRL-AR) and screened for antimicrobial susceptibility. The four *S*. Infantis isolates originated from broilers (*n* = 3) and from broiler meat sample (*n* = 1) (Supplementary Table 1).

The authors apologize for this error and state that this does not change the scientific conclusionsof the article in any way.

The original article has been updated.

## Conflict of interest statement

The authors declare that the research was conducted in the absence of any commercial or financial relationships that could be construed as a potential conflict of interest.

