# Supplementary material for: Colistin Resistance Mediated by mcr-1 in ESBL-Producing, Multidrug Resistant Salmonella Infantis in Broiler Chicken Industry, Italy (2016–2017)
Source: Front Microbiol. 2018 Aug 17;9:1880. doi: 10.3389/fmicb.2018.01880 (PMC6108180; doi:10.3389/fmicb.2018.01880)
Supplement: Supplementary file 1 [file Table_1.DOC]

**Supplementary Table 1:** Details of the 16 *S.* Infantis isolates selected for (SNP)-based phylogeny

| **Isolate ID** | **Year of isolation** | **Origin** | **Sample Context** | **Genetic Characteristics** | **ESC-R/ESC-S** |
| --- | --- | --- | --- | --- | --- |
| 06029746 IncI1 | 2006 | Broiler meat | Laboratory-based surveillance | *gyr*B wt, IncI1 | ESC-S |
| 07041415 pESI | 2007 | Broiler meat | Laboratory-based surveillance | *gyr*A (S83Y), pESI | ESC-S |
| 09051564-33 IncI1 | 2009 | Guinea fowl | Laboratory-based surveillance | *gyrA wt,* IncI1 | ESC-S |
| 09051564-79 IncI1 | 2009 | Guinea fowl | Laboratory-based surveillance | *gyr*A wt, IncI1 | ESC-S |
| 12037823-11 pESI | 2012 | Broiler chicken | National Control Programmes^1^ | *gyr*A (D87G), pESI (*bla*_CTX-M-1_) | ESC-R |
| 13002124-1 pESI | 2013 | Human | Laboratory-based surveillance | *gyr*A (D87G), pESI (*bla*_CTX-M-1_) | ESC-R |
| 13002124-34 pESI | 2013 | Broiler meat | Laboratory-based surveillance | *gyr*A (D87G), pESI (*bla*_CTX-M-1_) | ESC-R |
| 13017779-5 pESI | 2013 | Broiler chicken | National Control Programmes^1^ | *gyr*A (D87G), pESI (*bla*_CTX-M-1_) | ESC-R |
| 13065790-185 pESI | 2013 | Pig | Laboratory-based surveillance | *gyr*A (D87G), pESI (*bla*_CTX-M-1_) | ESC-R |
| 14026835 pESI | 2014 | Human | Laboratory-based surveillance | *gyr*A (D87G), pESI (*bla*_CTX-M-65_) | ESC-R |
| 14035093 pESI | 2014 | Human | Laboratory-based surveillance | *gyr*A (D87G), pESI (*bla*_CTX-M-1_) | ESC-R |
| 14057027-15 pESI | 2014 | Broiler meat | Laboratory-based surveillance | *gyr*A (D87G), pESI (*bla*_CTX-M-1_) | ESC-R |
| 16092401-41 pESI IncX4 | 2016 | Broiler chicken | National Control Programmes^1^ | *gyr*A (D87G), pESI (*bla*_CTX-M-1_), IncX4 (*mcr*-1.1) | ESC-R |
| 16092401-42 pESI IncX4 | 2016 | Broiler chicken | National Control Programmes^1^ | *gyr*A (D87G), pESI (*bla*_CTX-M-1_), IncX4 (*mcr*-1.1) | ESC-R |
| 16072017 pESI IncX4 | 2016 | Broiler chicken | National Monitoring^1^ | *gyr*A (D87G), pESI, IncX4 (*mcr*-1.1) | ESC-S |
| 17095712-68 pESI IncX4 | 2017 | Broiler meat | Laboratory-based surveillance | *gyr*A (D87G), pESI, IncX4 (*mcr*-1.1) | ESC-S |

wt= wild-type

ESC-R/ESC-S= extended-spectrum cephalosporin-resistant/extended-spectrum cephalosporin-susceptible

^1^Different epidemiological units (flocks)
