# Supplementary material for: Colistin Resistance Mediated by mcr-1 in ESBL-Producing, Multidrug Resistant Salmonella Infantis in Broiler Chicken Industry, Italy (2016–2017)
Source: Front Microbiol. 2018 Aug 17;9:1880. doi: 10.3389/fmicb.2018.01880 (PMC6108180; doi:10.3389/fmicb.2018.01880)
Supplement: Supplementary file 2 [file Table_2.DOC]

**Supplementary Table 2.**

List of reference sequences added to the CGE database and used in the BLASTN analysis

| **Gene** | **Function** | **Genbank accession number** |
| --- | --- | --- |
| qacEΔ | resistance to quaternary ammonium compounds | ASRF01000099 |
| backbone pESI | backbone pESI | ASRF01000099 |
| *k88* | k88-like fimbria | ASRF01000100 |
| *feaD* | usher | ASRF01000100 |
| *fim* | fimbria | ASRF01000100 |
| *ipf* | Infantis plasmid-encoded fimbria | ASRF01000100 |
| *irp2* | yersiniabactin biosynthetic protein | ASRF01000100 |
| *mer* | mercury(II) reductase | ASRF01000099 |
| *DNA_rep* | DNA replicase | ASRF01000099 |
| *ccdB* | CcdB toxin | ASRF01000100 |
| *ccdA* | CcdA antitoxin | ASRF01000100 |
| *pemK* | Programmed cell death toxin | ASRF01000100 |
| *pemI* | Programmed cell death antitoxin | ASRF01000100 |
| *hicA* | HicA antitoxin | KY120364 |
| *hicB* | HicB toxin | KY120364 |
| *trfA2* | Replication protein,long form | AM261760 |
| incP | Plasmid RK2 (fromE.coli) DNA with transposon (Tn*1723*) insertion sites | M20134.1 |
| incP | *oriV* IncP alpha plasmid pBS22 | AM261760 |
